# Food access, diet quality, and nutritional status of older adults during COVID-19: A scoping review

**DOI:** 10.1093/geroni/igab046.3393

**Published:** 2021-12-17

**Authors:** Emily Nicklett, Kimson Johnson, Lisa M Troy, Maitreyi Vartak, Ann Reiter

**Affiliations:** 1 The University of Texas at San Antonio, San Antonio, Texas, United States; 2 University of Michigan, Ann Arbor, Michigan, United States; 3 University of Massachusetts Amherst, University of Massachusetts Amherst, Massachusetts, United States

## Abstract

COVID-19 has imposed challenges for older adults to access food, particularly in minority, lower income, and rural communities. However, the impact of COVID-19 on food access, diet quality, and nutrition of diverse older adult populations has not been systematically assessed. The objective of this study is to examine changes in food access, diet quality, and nutritional status among older adults during the COVID-19 pandemic and the potential differential impacts of the COVID-19 pandemic on these nutrition-related outcomes using the framework of the socio-ecological model. An electronic search was conducted using three databases (PubMed, CINAHL, and Web of Science) on March 7, 2021. Original, peer-reviewed English-language studies published 10/1/2019-3/1/2021 were considered for which the age of participants was 50 years and older (average age range 50-98). In order to be considered, studies must have examined food access, food security, or nutrition constructs as an outcome. The initial search yielded 13,628 results, of which 9,145 were duplicates. Of the remaining 4,483 articles, 13 articles were in scope and therefore selected in the final analysis, which can be characterized as descriptive (n=5), analytical (n=6), and correlational (n=2). Studies were conducted among community-dwelling older adult populations (n=7) as well as those temporarily residing in hospital settings (n=6) in 10 countries. More research is needed to examine the impact of COVID-19 on food access/security and the differential barriers experienced by older adult populations.

